# Acute respiratory distress syndrome caused by *Chlamydia psittaci*: a case report and literature review

**DOI:** 10.3389/fmed.2024.1418241

**Published:** 2024-12-03

**Authors:** Yan Zhou, Ya Zou, Lu Zhou, Hua Wei, Yong-Wei Zou, Xi-Rui Guo, Yong-Qin Ye, Na Li, Yun Lu

**Affiliations:** ^1^Department of Pharmacy, Chengdu Second People’s Hospital, Chengdu, Sichuan, China; ^2^Department of Respiratory and Critical Care Medicine, Chengdu Second People’s Hospital, Chengdu, Sichuan, China; ^3^Department of Pharmacy, Central People’s Hospital of Zhanjiang, Zhanjiang, Guangdong, China

**Keywords:** *Chlamydia psittaci*, acute respiratory distress syndrome, epidemiological investigation, metagenomic next-generation sequencing, case report

## Abstract

**Background:**

Psittacosis is a zoonotic disease with a low incidence rate and a lack of specificity in clinical manifestations, making it prone to be missed, misdiagnosed, and even cause delayed treatment for patients. Metagenomic next-generation sequencing (mNGS) was successfully performed for the diagnosis of a young patient with psittacosis progressing to acute respiratory distress syndrome (ARDS), and precisely targeted antibiotic treatment was promptly administered. Additionally, a comprehensive review was conducted on 68 cases of psittacosis complicated with ARDS, with the goal of improving the clinical awareness of this disease.

**Case presentation:**

This study reports a 37-year-old young female who was infected with *Chlamydia psittaci* (*C. psittaci*) after contact with parrots and eventually developed ARDS. The patient initially developed fever and sore throat, followed by cough and expectoration. Despite receiving empirical anti-infection treatment, the condition continued to progress rapidly, and severe dyspnea developed within a short period of time. She was subsequently transferred to the intensive care unit (ICU) and underwent tracheal intubation and mechanical ventilation due to acute respiratory failure. After the DNA sequence of *C. psittaci* in bronchoalveolar lavage fluid (BALF) was detected through mNGS, the patient received targeted antibiotic treatment with doxycycline and moxifloxacin, and her clinical symptoms gradually improved.

**Conclusion:**

Epidemiological investigations and the application of mNGS are crucial for the early identification and diagnosis of psittacosis. For suspected psittacosis patients, the application of mNGS technology could promote early identification of pathogens and targeted antimicrobial therapy, which might improve patient prognosis. In addition, young psittacosis patients without underlying disease should also be vigilant about the possibility of developing severe cases.

## 1 Introduction

Psittacosis, also known as ornithosis, is caused by infection with *Chlamydia psittaci* (*C. psittaci*). Moreover, *C. psittaci* is a gram-negative and specialized intracellular parasitic pathogen, which is also one of the species in the Chlamydia family that is pathogenic to humans or animals (1). It can directly or indirectly infect the host, leading to the onset of psittacosis. Specifically, psittacosis spreads mainly between birds and can be transmitted to humans through birds or other animals in specific situations (2). In addition, it can also be transmitted from human to human (3, 4). The interpersonal transmission of *C. psittaci* is regarded as a newly emerging public health risk, especially for healthcare workers and their close contacts (5). Psittacosis mainly manifests as pneumonia, but only approximately 1% of community-acquired pneumonia is caused by psittacosis (6). Although the incidence rate is relatively low, *C. psittaci* pneumonia accounts for a high proportion (8%) of severe community-acquired pneumonia cases (7). The asymptomatic spread of *C. psittaci* and inadequate laboratory detection techniques may lead to a serious underestimation of the current global number of psittacosis cases. In recent years, with the migration of migratory birds and the increase in the number of pet birds, reports of animals and humans infected with *C. psittaci* have increased annually, indicating that *C. psittaci* poses a potential threat to animal husbandry and human health. However, owing to nonspecific clinical symptoms, the lack of laboratory testing and diagnostic tools, and insufficient clinical understanding of the disease, psittacosis is often ignored in the diagnosis process, leading to delayed treatment in clinical practice, which undoubtedly increases the health burden of psittacosis worldwide.

Unfortunately, there is currently no effective vaccine to prevent psittacosis. If not treated promptly after infection, a critical illness may develop in a short period of time, seriously affecting the patient’s prognosis. Rapid and accurate identification of clinical pathogens and timely targeted antibiotic treatment are key strategies for the successful treatment of psittacosis. With the application of metagenomic next-generation sequencing (mNGS) technology in the clinic, many atypical pathogens can be validly detected. An increasing number of cases of *C. psittaci* pneumonia have been clearly diagnosed, providing timely and effective targeted treatment for clinical practice.

Previous studies have shown limited case reports of psittacosis complicated with acute respiratory distress syndrome (ARDS). However, the hospitalization mortality rate of severe ARDS patients is relatively high, ranging from 26 to 60%, posing a serious threat to public life and health safety (8, 9). Herein, this is a report of a young female patient infected with *C. psittaci* who was diagnosed by mNGS and progressed to ARDS. In addition, we summarized the epidemiology, clinical manifestations, laboratory data, complications and treatment strategies of 68 patients with severe *C. psittaci* pneumonia complicated with ARDS to improve the clinical understanding of the diagnosis and treatment of this disease.

## 2 Case presentation

A 37-year-old female patient was admitted to the Department of Respiratory and Critical Care Medicine at Chengdu Second People’s Hospital on February 27, 2023, due to cough and expectoration. The patient presented with sore throat and fever (39.3°C) 3 days before admission. The patient subsequently took ceftizoxime (0.2 g po bid) and diclofenac sodium sustained-release tablets (0.1 g po qd), but the above symptoms did not improve. The patient has good past health without personal or family history. Epidemiological investigations revealed that the patient purchased two parrots from a pet store on January 25, 2023. A parrot died on February 19, 2023, and the patient cleared its body and feces.

**Figure 1:**
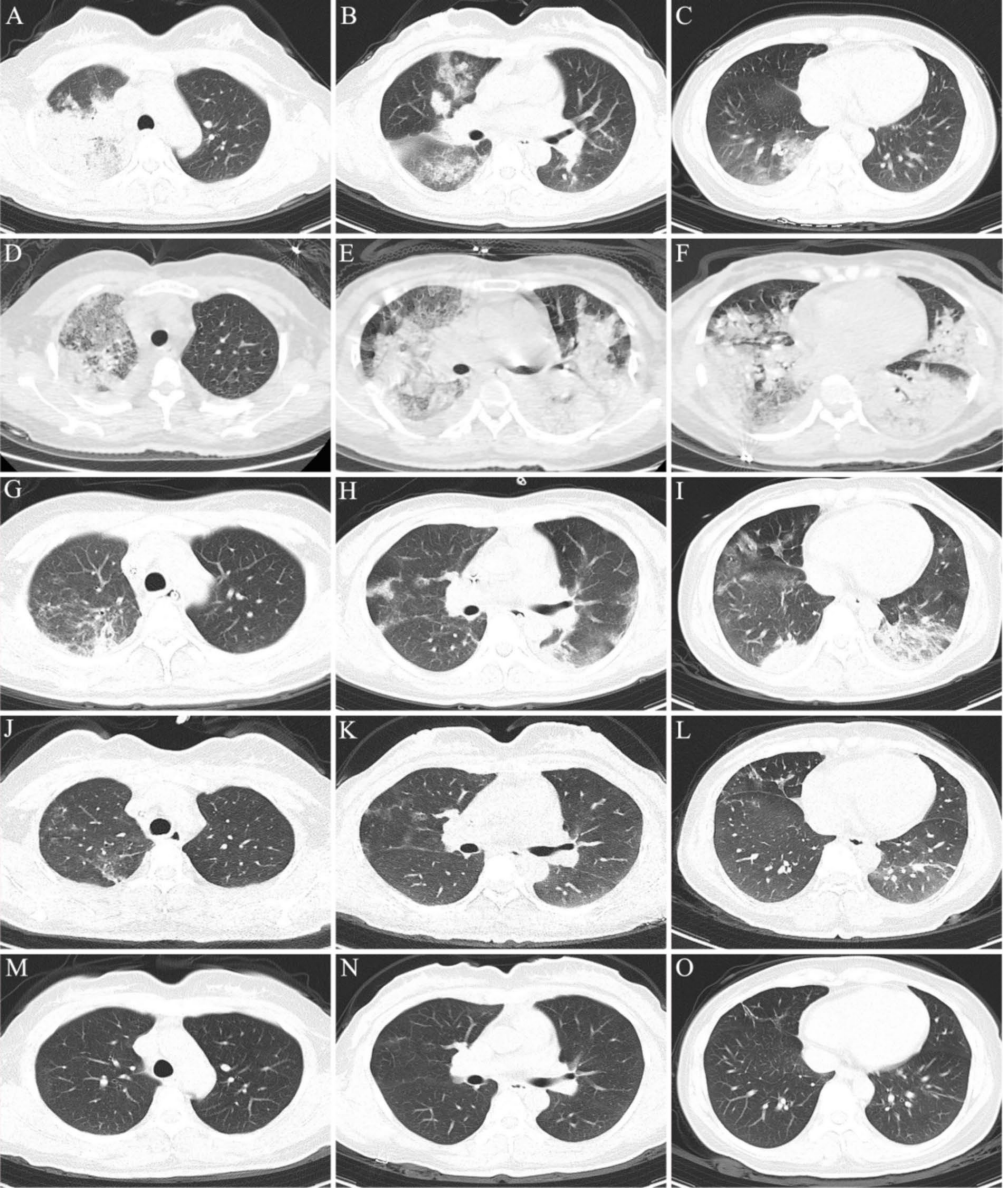
Chest CT scan of the patient during hospitalization and follow-up. (A–C) On the 1th day after admission; (D–F) on the 6th day after admission; (G–I) on the 12th day after admission; (J–L) on the day of discharge; (M–O) on the 17th day of follow-up after discharge.

Physical examination on admission revealed that body temperature, heart rate (HR), respiratory rate, and blood pressure were 39.3°C, 99 beats/min, 22 beats/min, and 97/63 mmHg, respectively. Initial lung computed tomography (CT) revealed multiple patchy and nodular high-density shadows in both lungs, mainly involving the upper and lower lobes of the right lung. It was partially consolidated with right pleural effusion and mediastinal lymph node enlargement, as shown in Figures 1A–C. In addition, the patient was fully conscious and experienced mild shortness of breath. Chest auscultation revealed wet rales in both lungs. Laboratory tests on admission revealed a white blood cell (WBC) count of 6.4 × 10^9^·L^−1^, neutrophils (NEUT) count of 5.5 × 10^9^·L^−1^, C-reactive protein (CRP) of 88.8 mg/L, procalcitonin (PCT) of 0.12 ng/mL, alanine aminotransferase (ALT) of 123 U/L, aspartate aminotransferase (AST) of 113 U/L, alkaline phosphatase (ALP) of 246 U/L, and gamma-glutamyl transpeptidase (GGT) of 171 U/L. Arterial blood gas analysis (ABGA) revealed that the pH, oxygen partial pressure (PaO_2_), carbon dioxide partial pressure (PaCO_2_), oxygenation index (OI), and HCO3^−^ and K^+^ concentrations were 7.47, 30.7 mmHg, 38.2 mmHg, 93.2 mmHg, 27.6 mmol/L and 3.1 mmol/L, respectively. The patient was diagnosed with community-acquired pneumonia and type I respiratory failure, who received oxygen therapy through a facemask at a rate of 4 L/min and a fraction of inspired oxygen (FiO2) 37%. In addition, the patient also received treatment with polyene phosphatidylcholine capsules (456 mg po tid), diclofenac sodium sustained-release tablets (0.1 g po qd), administered cefazoxime (2.25 g ivgtt q8h), and nebulized inhalation of acetylcysteine (0.3 g inh tid).

On the 5th day after admission, the patient was in a poor mental state and had a fever with a maximum body temperature above 40°C, lip cyanosis, and a respiratory rate of more than 30 beats/min. Breathing sounds thicker, and wet rales in both lungs were audible. The oxygen saturation (SpO_2_) was 60 ~ 70%, and the patient immediately received noninvasive mechanical ventilation (ST mode, IPAP 15cmH2O, EPAP 4cmH2O, FiO_2_ 40%). The sputum and blood culture results for typical bacteria and fungi, as well as the relevant tests for *Mycobacterium tuberculosis* were negative. Serological tests for novel coronavirus (2019-nCoV) nucleic acid, influenza virus nucleic acid, (1,3)-*β*-D-glucan (BDG), galactomannan (GM), the capsular polysaccharide antigen of *Cryptococcus,* and IgM antibodies against respiratory pathogens were negative. In addition, the serum antinuclear antibodies (ANAs), antineutrophil autoantibodies, and immunoglobulin concentrations were within the normal ranges.

On the 6th day after admission, the patient developed a high fever with a maximum body temperature of 40.3°C, accompanied by drowsiness and confusion. Moreover, there was significant shortness of breath, with a respiratory rate of approximately 40 beats/min. SpO_2_ continued to decrease and could not be maintained at normal levels after slight activity. Arterial blood gas analysis (ABGA) revealed that the pH, PaO_2_, PaCO_2_, SpO_2_, PaO_2_/FiO_2_, SpO_2_/FiO_2_, and PaO_2_/FiO_2_ were 7.53, 54.0 mmHg, 33.9 mmHg, 88.6%, 90.0 mmHg, 147.7, and < 100 mmHg, respectively. In accordance with the ‘Berlin Definition’, the patient was diagnosed with severe ARDS (10). Chest CT revealed multiple plaques and high-density shadows in both lungs with bronchograms, partial consolidation, and partial interstitial involvement. The infection was significantly aggravated, with a slight increase in pleural effusion, as shown in Figures 1D–F. The patient subsequently developed refractory respiratory failure and was transferred to the intensive care unit (ICU), where she received tracheal intubation and invasive mechanical ventilation (V-A/C mode, VTE 360 mL, PEEP 8 cmH2O, FiO_2_ 100%). To reduce the risk of ventilator-induced lung injury (VILI) and increase the effective alveolar ventilation area, some lung-protective ventilation strategies, such as low tidal volume (6 mL/kg), low plateau pressure (20 cmH2O), high end-expiratory pressure (8 cmH2O), and prone position ventilation have been implemented. The antibiotics were subsequently adjusted to cefoperazone/sulbactam (3 g ivgtt q8h) combined with moxifloxacin (0.4 g ivgtt qd) for treatment. Owing to the rapid progression of the disease, vancomycin was added, but due to the redness of the patient’s skin after intravenous infusion, it was immediately adjusted to linezolid (0.6 g ivgtt q12h). Moreover, samples of the bronchoalveolar lavage fluid (BALF) were collected and tested via mNGS for etiological diagnosis.

**Figure 2:**
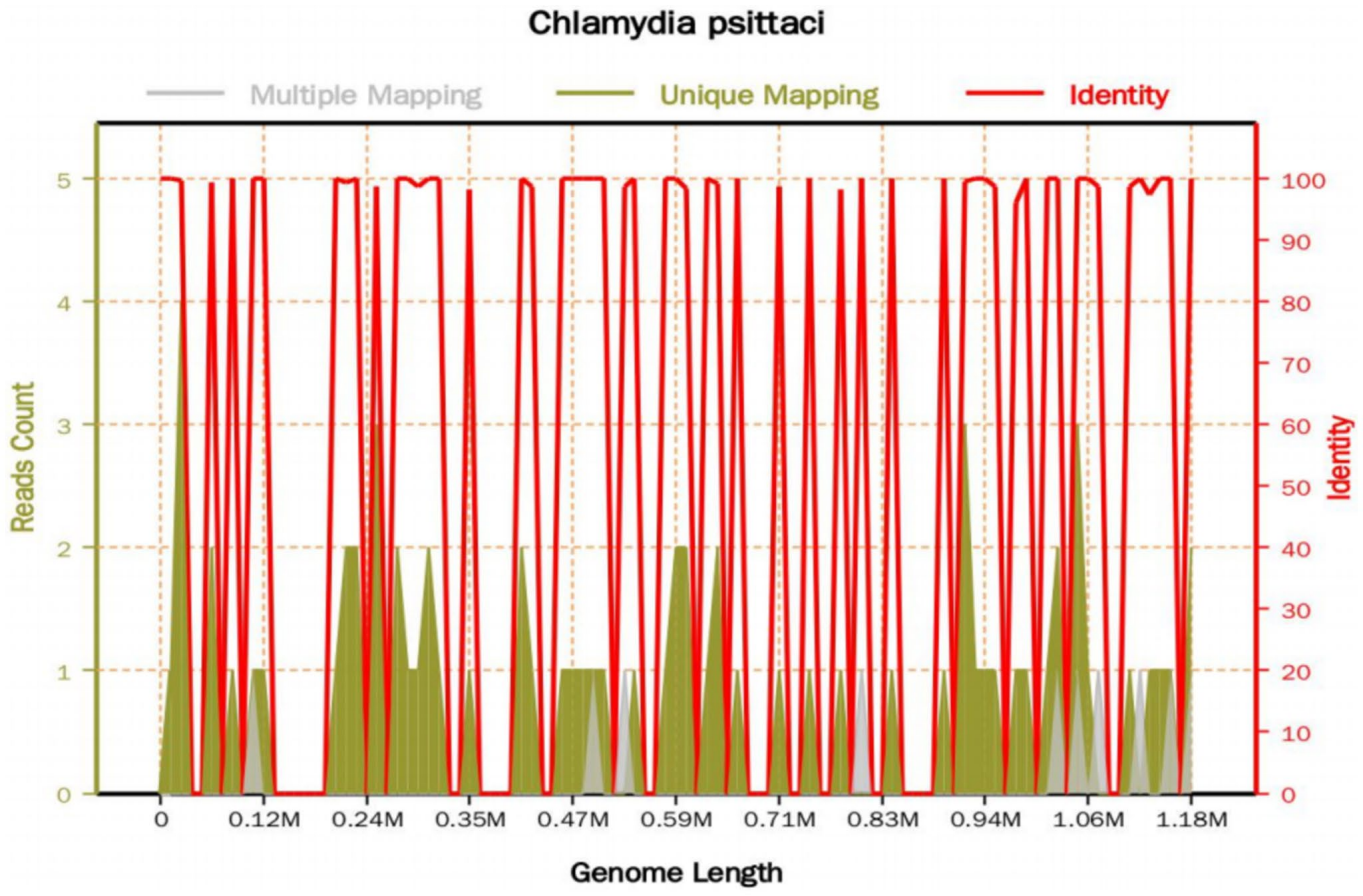
The mNGS sequencing results of the BALF sample. The total base number of the detected genome is 1,179,220 (bp), of which the total length covered by the *C. psittaci* sequence is 5,764 (bp), with a coverage of 0.488798% and an average depth of 1.04X. A total of 70 sequence reads corresponding to *C. psittaci* were identified.

**Figure 3:**
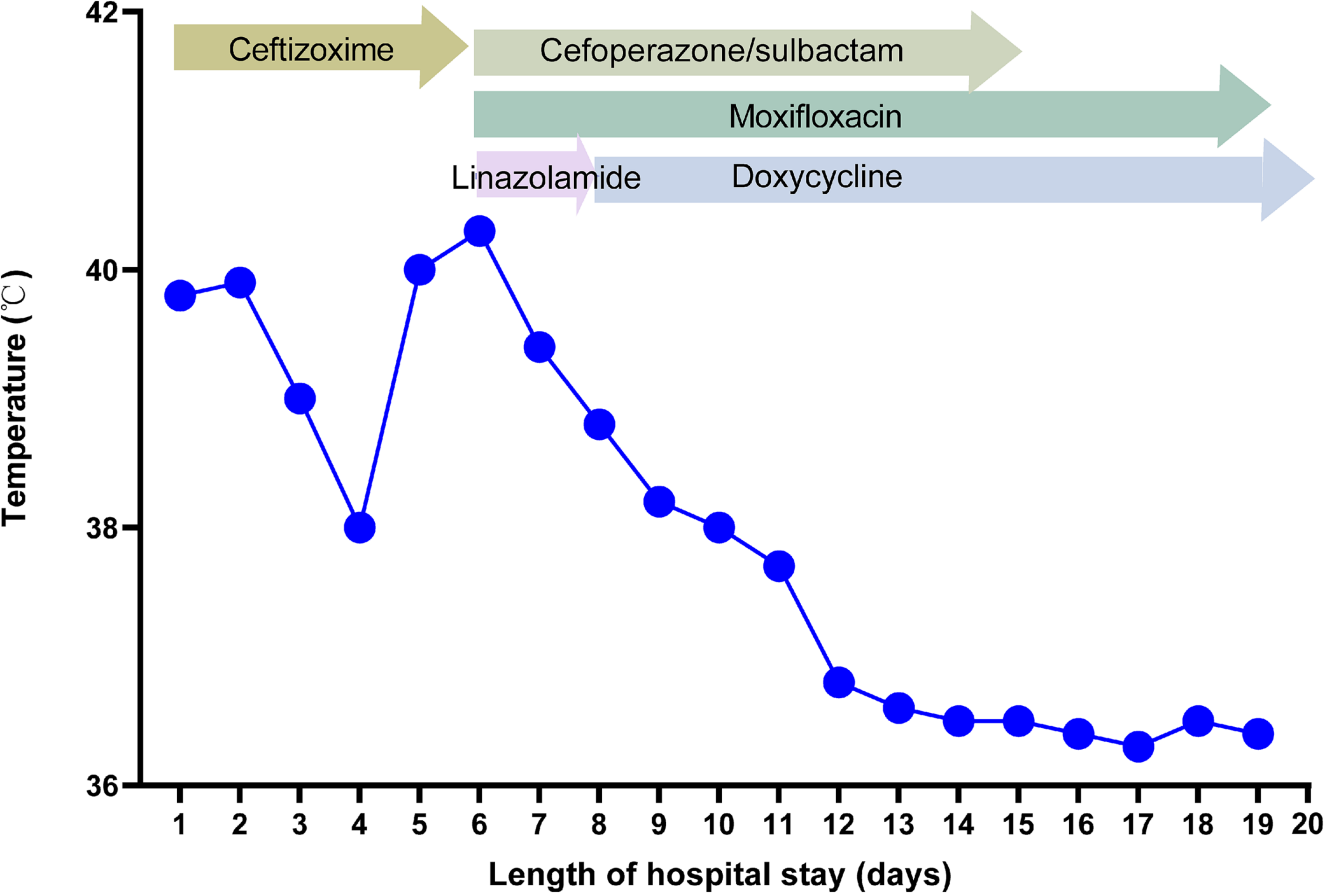
Body temperature and antibiotic treatment during hospitalization.

**Figure 4:**
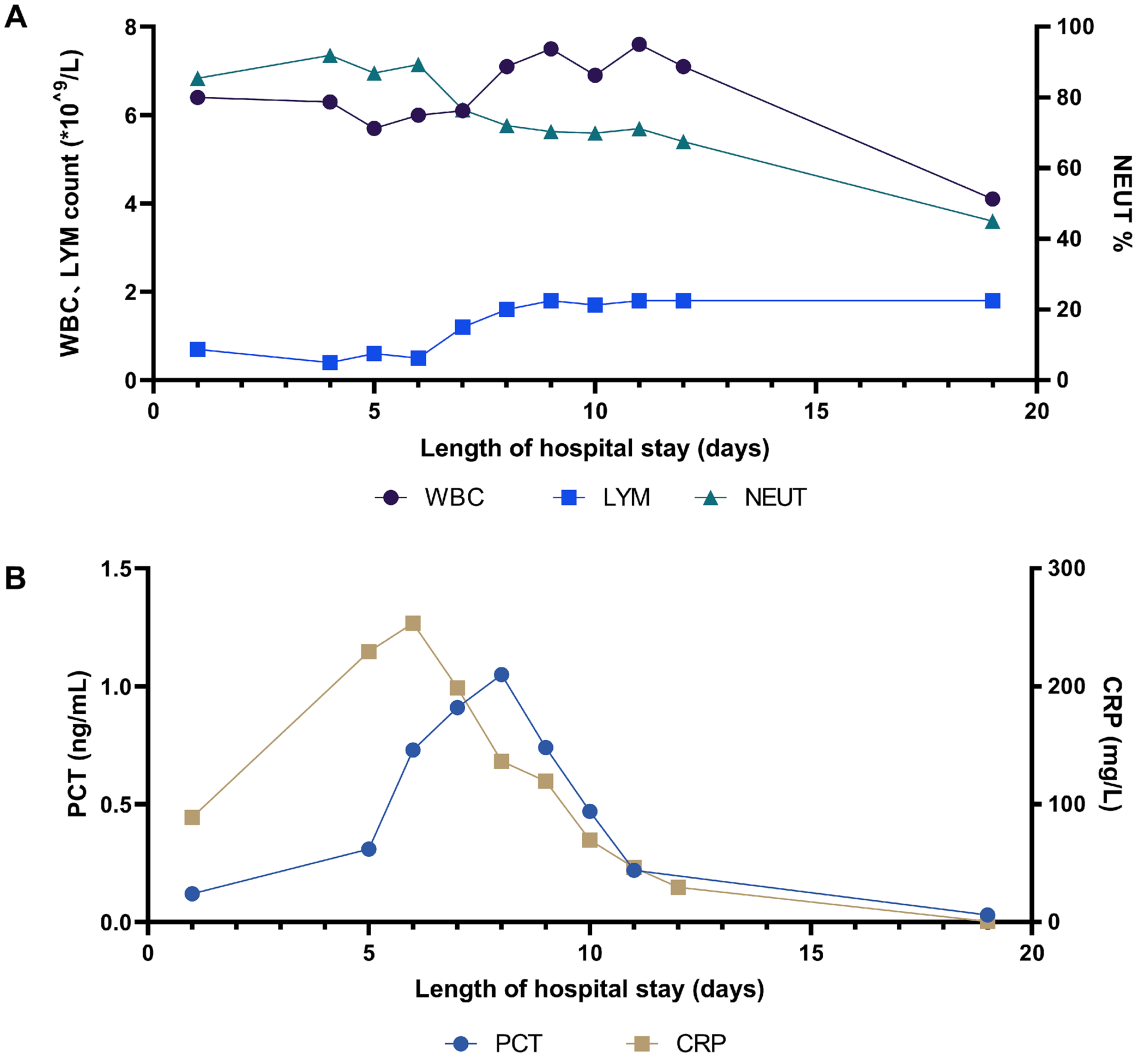
Change in (A) WBC count, LYM count and NEUT%, and (B) PCT and CRP during hospitalization.

**Figure 5:**
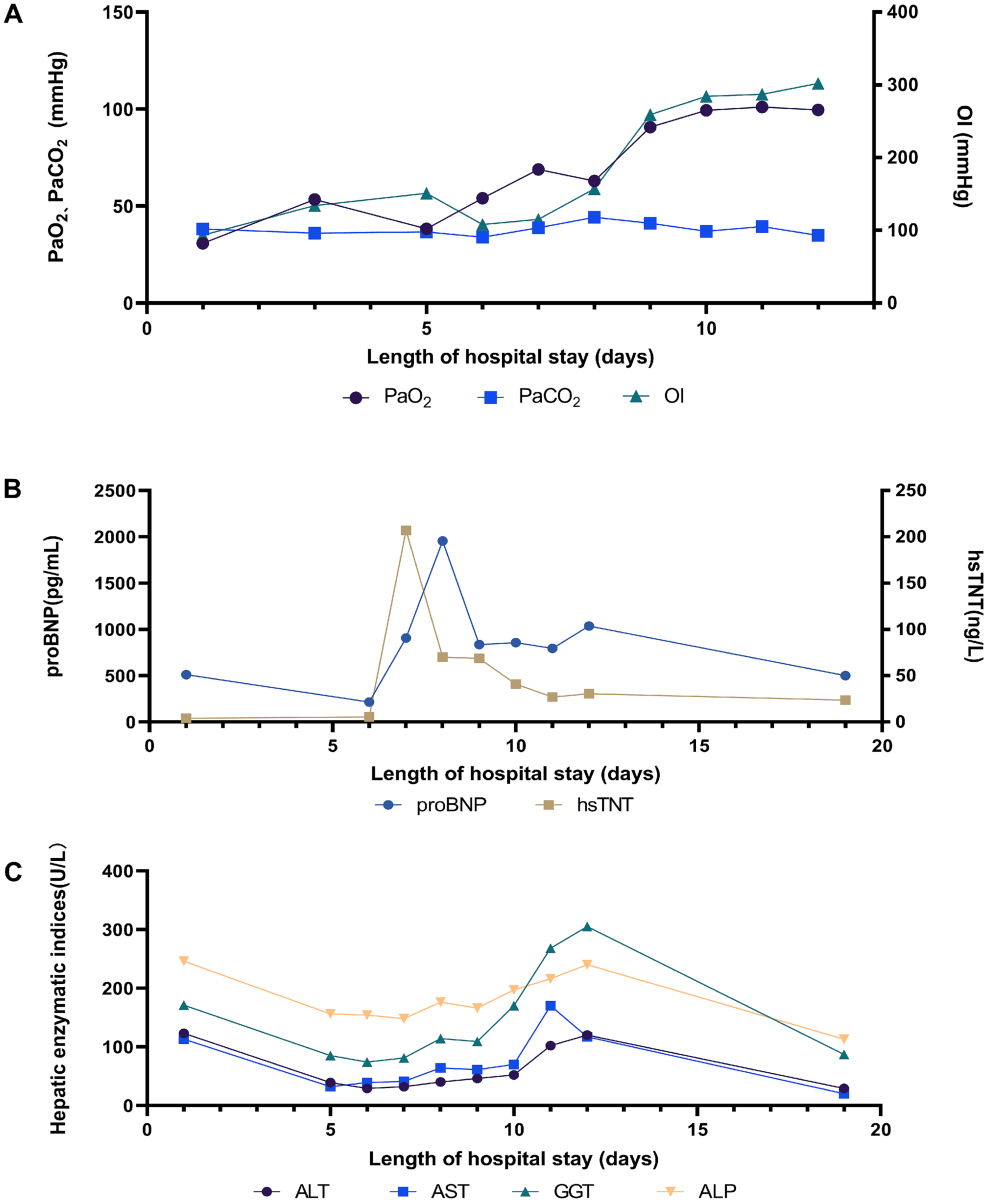
Change in (A) lung, (B) heart, and (C) liver related indicators during hospitalization.

On the 8th day after admission, the BALF results were reported, and 70 sequence reads corresponding to *C. psittaci* were identified, with a relative abundance of 0.49%, as shown in Figure 2. Considering the recent contact history with parrots, the patient was finally diagnosed with *C. psittaci* pneumonia. The patient was subsequently adjusted to receive a combination therapy of targeted antibiotics: moxifloxacin (0.4 g ivgtt qd) and doxycycline (0.1 g po q12h). After effective treatment, the patient’s clinical symptoms gradually improved. Owing to improvements in respiratory function, invasive mechanical ventilation was withdrawn after 5 days of clinical application. On the 11th day after admission, echocardiography revealed a decrease in ventricular septal contractile activity, mild pericardial effusion, and a decrease in the left ventricular ejection fraction (LVEF: 48%). On the 12th day after admission, chest CT showed significant absorption of pulmonary inflammation and reduced pleural effusion compared with before, as shown in Figures 1G–I. Considering the absence of a history of underlying cardiac disease and no significant abnormalities were observed on the electrocardiogram after admission, it was speculated that the patient’s cardiac changes were related to infection with *C. psittaci*. On the 13th day after admission, the patient’s condition improved with temperature returned to normal (Figure 3) and she was transferred to a general ward for subsequent treatment. Owing to timely and accurate targeted antibiotic treatment, the patient recovered (Figures 4, 5) and was discharged 7 days later. On the day of discharge, chest CT revealed further absorption of pulmonary inflammation, and no pleural effusion was observed, as shown in Figures 1J–L. The patient continued to receive doxycycline treatment outside the hospital for 12 days. On the 17th day of follow-up after discharge, a chest CT revealed a small amount of patchy and linear high-density shadows, as shown in Figures 1M–O, and the rest completely recovered.

## 3 Literature review

**Figure 6:**
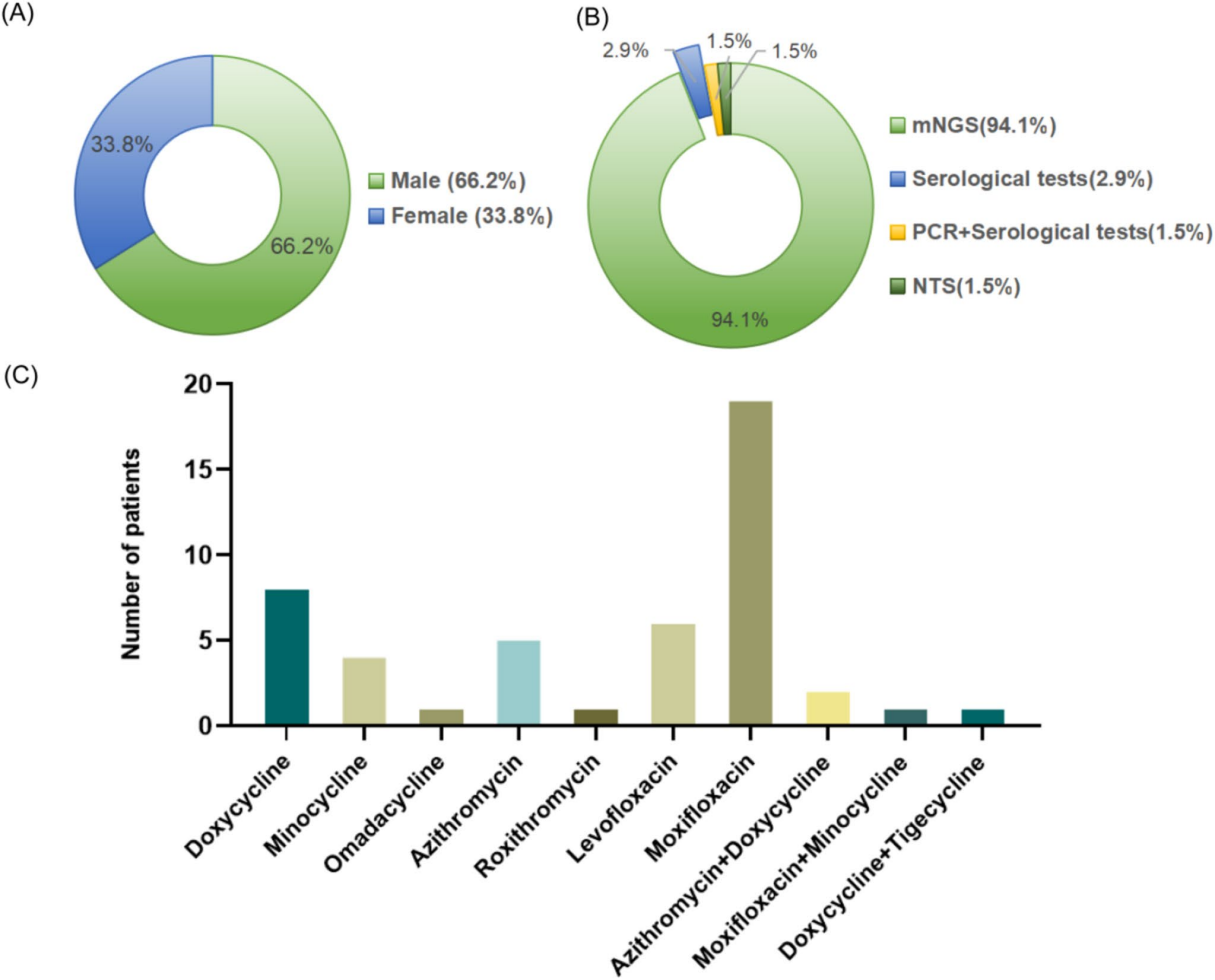
Gender (A), pathogen detection methods (B), and anti-infective drugs after diagnosis of psittacosis (C) of the 68 patients in our literature review.

We searched the PubMed and Embase databases for articles on ARDS caused by *C. psittaci* published before January 31, 2024. The search strategies were “psittacosis” and “acute respiratory distress syndrome,” and a total of 79 articles were retrieved. Excluding duplicate studies or those with incomplete original data, a total of 9 articles were included for further study, with a total of 68 patients, including 45 males (66.2%) and 23 females (33.8%), as shown in Figure 6A. Detailed information is shown in Table 1. Among them, 64 cases were detected for *C. psittaci* through mNGS, 1 case was detected through nanopore targeted sequencing (NTS), 2 cases were confirmed through serological testing, and 1 case was confirmed through both serological testing and polymerase chain reaction (PCR) (Figure 6B). In particular, the majority of cases (*n* = 45, 66.2%) had a history of contact with birds and poultry, or exposure to the environment. Forty-six patients (67.6%) were reported to have underlying diseases, including hypertension, diabetes, cirrhosis and hepatitis B. In addition, 62 patients (91.2%) had fever upon admission. With the progression of the disease, most patients also experienced organ damage such as the liver, kidneys, heart, digestive system, and nervous system, in addition to developing into ARDS. The liver was the most commonly affected, including 58 cases (85.3%), and liver function involvement was reported to be unrelated to the underlying diseases of hepatitis B or cirrhosis. After the corresponding treatment, their liver function recovered. All patients received different ventilation methods to improve hypoxia. Notably, it was found that renal dysfunction might be a high-risk factor for patients with psittacosis complicated with ARDS, as the proportion of renal dysfunction in the deceased group was significantly higher than that in the survived group (83.3% *vs* 12.9%, *p* = 0.001) through Fisher’s exact test (Supplementary Table S1). Sixteen patients (23.5%) received positive pressure ventilation (PPV), and six patients (8.8%) received extracorporeal membrane oxygenation (ECMO) or extracorporeal lung assist (ELA) replacement therapy because of the difficulties in maintaining oxygenation during mechanical ventilation treatment. After being diagnosed with *C. psittaci* pneumonia, all patients received a single or combination therapy of tetracyclines (doxycycline, minocycline, tigecycline or omadacycline), quinolones (levofloxacin or moxifloxacin), or macrolides (azithromycin or roxithromycin), as shown in Figure 6C. Finally, 62 cases (91.2%) recovered and 6 cases (8.8%) died.

**Table 1:** Summary of case series and case report of ARDS caused by *C. psittaci*.

Author	Reported area/time	Case number	Methods	Gender	Age (years)	Underlying disease	History of contact	Clinical symptoms on the day of admission	Complications	Empirical anti-infective drugs	Anti-infective drugs after diagnosis of psittacosis	Ventilation methods	Clinical outcome
Marchese et al. (24)	Italy /2023	1	PCR and serological tests	Male	3.5	/	Parrot	Cough and dyspnea	/	Ceftriaxon, Teicoplanin	Doxycycline	VAM + IOT	Recovered
Wichert et al. (49)	Germany /2000	1	Serological tests	Male	47	/	Poultry	Fever, cough, expectoration, confusion, myalgia, and dehydration	Renal dysfunction, gastrointestinal symptoms, and neurological symptoms	The initial treatment was ceftibuten, and then changed to erythromycin, ciprofloxacin, and rifampicin.	Doxycycline	IMV + ELA	Recovered
Yang et al. (50)	China/2021	27	mNGS	Male (19); Female (8)	Average age of 60 (range:35–8)	Hypertension (10 cases); Liver cirrhosis (11 cases); Diabetes (1 case); Pneumoconiosis (1 case); Gout (1 case)	Pigeon (4 cases); Poultry (6 cases)	Fever (23 cases); Dyspnea (22 cases); Cough (19 cases); Headache (10 cases); Diarrhea (3 cases); Backache (1 case)	Liver dysfunction (27 cases); Neurological symptoms (9 cases); Renal dysfunction (4 cases); Hyponatremia (19 cases)	Not recorded	Quinolones (17 cases); Tetracyclines (6 cases); Azithromycin (5 cases); Azithromycin+Doxycycline (1 case)	NIPPV (13 cases); IMV + PPV (11 cases); The initial treatment was IMV + PPV, and then changed to ECMO (3 cases)	Recovered (23 cases); Died (4 cases)
Wang et al. (18)	China /2021	1	NTS	Female	48	Hemorrhoids, gastric ulcers and cervical spondylosis	Environmental exposure	Fever, diarrhea, chest pain, weakness, and syncope	Renal dysfunction	The initial treatment was ceftazidime, then changed to meropenem + ganciclovir, and finally changed to azithromycin.	Doxycycline	The initial treatment was IMV, and then changed to ECMO	Recovered
Ferreira et al. (51)	Brazil /2017	1	Serological tests	Female	19	/	Parrot	Fever, cough, dyspnea, and chest pain	/	Clarithromycin, Ceftriaxone	Doxycycline	Mask oxygen inhalation	Recovered
Yang et al. (52)	China /2023	1	mNGS	Female	51	/	/	Lisp., lower limb weakness, dizziness, nausea, and confusion	Pulmonary embolism	The initial treatment was piperacillin/tazobactam, and then changed to cefoperazone/sulbactam and levofloxacin.	Doxycycline+Azithromycin	The initial treatment was HFNC, then changed to IMV, and finnally changed to ECMO+PPV.	Recovered
Zhao et al. (25)	China /2022	10	mNGS	Male (6); Female (4)	Average age of 57 (range: 29–80)	Diabetes and coronary heart disease (1 case); Diabetes and hypertension (1 case); Diabetes, hypertension and cerebral infarction (1 case); Chronic obstructive pulmonary disease (1 case); Hypertension (1 case); Previous pulmonary tuberculosis (1 case)	Parrot (2 cases); Pigeons (1 case); Chick (1 case); Mynah (1 case); Poultry (1 case)	Fever and dyspnea (10 cases); Hemoptysis (4 cases)	Hepatic dysfunction (7 cases); Renal disorder (3 cases); Cardiac damage (5 cases); Hyponatremia (9 cases); Gastrointestinal symptoms (2 cases); Neurological symptoms (2 cases)	Not recorded	Moxifloxacin (18 cases); Minocycline (1 case); Azithromycin (1 case)	IMV (3 cases); NIPPV (2 cases); The initial treatment was NIPPV, and then changed to IMV (2 cases); HFNC (2 cases); The initial treatment was HFNC, and then changed to IMV (1 case); PPV (5 cases); PPV + NIPPV or HFNC (3 cases)	Recovered
Wang et al. (44)	China /2024	16	mNGS	Male (11); Femal e (5)	Average age of 65 (range: 39–76)	Hypertension (4 cases); Diabetes (1 case); Chronic obstructive pulmonary disease (1 case); Hepatitis B (1 case); Hypertension and diabetes (1 case); Diabetes and hepatitis B (1 case)	Parrot (10 cases); Pigeons (3 cases); Chickens and ducks (3 cases);	Fever (16 cases); Dyspnea (14 cases); Cough (11 cases); Shiver and headache (6 cases); Myalgia (7 cases)	Hepatic dysfunction (15 cases); Cardiac damage (14 cases); Renal dysfunction (4 cases); Pulmonary embolism (7 cases)	*β*-lactams/carbapenems ±quinolones	Omadacycline	IMV (10 cases); NIPPV (6 cases); Combination therapy of PPV (7 cases)	Recovered (14 cases); Died (2 cases)
Tang et al. (53)	China /2022	10	mNGS	Male (6); Female (4)	Average age of 62.9 (range: 46–74)	Hypertension (2 cases); Hepatitis B (2 cases); Diabetes and hypertension (2 cases)	Parrot (3 cases); Chickens and ducks (5 cases); Pigeons (2 cases);	Fever, weakness, and poor appetite (10 cases); Cough (4 cases); Myalgia (3 cases); Headache (2 cases); Chills (1 case)	Hepatic dysfunction (9 cases); Hypokalemia (7 cases)	Monotherapy or combination therapy of *β*-lactams/quinolones/carbapenems/antifungal agents /antiviral drugs	Moxifloxacin (3 cases); Doxycycline (2 cases); Minocycline (2 cases); Tigecycline (1 case); The initial treatment was roxithromycin, and changed to doxycycline (1 case); The initial treatment was moxifloxacin+minocycline, and changed to doxycycline+tigecycline (1 case)	IMV (cases); NIPPV (4 cases); HFNC (4 cases)	Recovered

VAM, mechanical assisted ventilation; IOT, oro-tracheal intubation; IMV, invasive mechanical ventilation; ELA, extracorporeal lung assist; NIPPV, noninvasive positive pressure ventilation; PPV, prone position ventilation; NTS, nanopore targeted sequencin; ECMO, extracorporeal membrane oxygenation; HFNC, high-flow nasal cannula.

## 4 Discussion

Psittacosis has erupted and spread in multiple regions worldwide (11, 12), with an increasing trend annually (5). Due to its potential to cause collective infection and illness during the epidemic, its infectivity and pathogenicity cannot be ignored, as it poses a significant threat to public health. Failure to diagnose diseases or identify pathogens in a timely manner could lead to delayed usage of effective antibiotics, resulting in high mortality rates in severe cases. Although there has been an increase in cases of psittacosis in recent years, psittacosis is still considered a rare disease, and there are still few reports on cases of ARDS caused by psittacosis. In addition, due to the diverse clinical manifestations and insufficient clinical understanding of the disease, it is often misdiagnosed or missed. Therefore, it is necessary to report and review cases of ARDS caused by psittacosis to promote the clinical understanding and management of the disease.

*Chlamydia psittaci* is a recognized zoonotic pathogen that is infectious to birds and poultry such as parrots, pigeons, chickens, and ducks, as well as mammals like horses, sheep, and cows. It can be transmitted to humans through the urine, feces, and other excreta of infected animals. In recent years, cases of human-to-human transmission have also been reported. The incubation period of psittacosis is usually 5 to 14 days, which may last up to 1 month (13). Patients often have a history of contact with birds and poultry (14, 15). Cleaning wild bird feeders or direct contact with feces contaminated with *C. psittaci* are considered important risk factors for infection (16). In addition, indirect or environmental contact with *C. psittaci* is also associated with infection with psittacosis (2, 17). In this report, the patient had contact with dead parrots before the onset of psittacosis, so dead parrots were highly likely to be the source of the pathogen. Our literature review reported a total of 68 cases, of which 44 had contact with birds or poultry. In addition, 1 patient had no clear history of contact, but lived in a community with large forests and birds (18). Therefore, in addition to inquiring about the patient’s direct contact history, it is also necessary to conduct a detailed investigation of the patient’s travel history, residential history, and environmental exposure, which may provide favorable clues for clinical diagnosis. However, patients without a clear history of contact are also common and cannot be ruled out.

Psittacosis is common in middle-aged and elderly people, with more males than females (19), and a lower risk of infection in children (20, 21). Psittacosis during pregnancy is rare, but it is highly likely to cause illness and death in pregnant women and fetuses (22, 23). According to reports, a 3.5-year-old child with *C. psittaci*-induced ARDS improved after treatment (24), whereas a pregnant woman discovered fetal death 2 days after undergoing tracheal intubation (25). Additionally, some reports have suggested that age (>65 year) and males are risk factors for severe *C. psittaci* pneumonia (19). This study indicates that among the cases with *C. psittaci*-induced ARDS, male patients accounted for a relatively large proportion, mainly middle-aged and elderly patients, which is consistent with previous reports. However, in this study, we reported a healthy young woman who eventually progressed to ARDS. Therefore, young women without underlying disease should also be vigilant about the possibility of developing into severe illness.

*Chlamydia psittaci* mainly infects the mucosal surface of human and animal hosts, causing various diseases (26). Initial replication begins in respiratory mucosal epithelial cells and macrophages, resulting in sepsis. Then, replication is carried out in epithelial cells and parenchymal tissues of the whole body (27), and various organs and systems may be affected. The clinical manifestations are diverse, and the severity of the disease varies from asymptomatic to severe (28). The respiratory system is most commonly affected, and almost all patients experience fever (26). Symptoms usually appear suddenly, and some patients may experience dyspnea. Severe cases may progress to ARDS (18), multiple organ dysfunction syndrome (MODS) (29, 30), and even death. Most patients also experience fatigue, muscle pain, central nervous system symptoms, and gastrointestinal symptoms (19). Notably, ARDS is usually associated with high mortality rates. An epidemiological study of ICU patients from 50 countries revealed that the incidence of ARDS was 10.4% of ICU admissions, and the hospital mortality rate was 46.1% for those with severe ARDS (8). Patients with psittacosis commonly progress to ARDS. A retrospective multicenter study involving 75 patients with psittacosis revealed that over half of the patients (41/74, 55.4%) developed ARDS, with 6 deaths and a mortality rate of 14.6% (31). However, owing to the limited sample scale, the overall incidence rate and mortality of ARDS caused by psittacosis still needs to be further determined through large-scale researches. Compared with general ARDS treatment strategies, timely use of antibiotics that can effectively inhibit *C. psittaci* is crucial for targeted therapy of psittacosis-related ARDS, in addition to respiratory support, nutritional support, fluid management, and symptomatic treatments such as glucocorticoids, sedatives, and muscle relaxants.

In the case reported in this study, the patient presented with fever as the primary manifestation, followed by cough, expectoration, and chills during the course of the disease. Her respiratory condition deteriorated rapidly, with dyspnea and ARDS, so she received mechanical ventilation. The pathogen was subsequently identified through mNGS, and timely treatment was provided. In addition to respiratory involvement, the liver and heart were also affected, which might be related to *C. psittaci* invading the liver and heart. In the literature review of this study, all patients with *C. psittaci* pneumonia who developed ARDS received oxygen therapy, severe cases received mechanical ventilation, and when NIPPV and PPV were insufficient to increase blood oxygen saturation, ECMO treatment was received. Almost all patients were diagnosed with ARDS combined with septic shock, and severe cases progressed to MODS. Among the 68 patients, 6 (8.8%) died due to multiple organ failure caused by secondary infection. Respiratory support, especially mechanical ventilation, plays an important role in the treatment of ARDS patients. Thus, the adoption of lung- protective ventilation strategies is recommended for all ARDS patients (32). Owing to the high metabolic status of ARDS patients, appropriate nutritional support should be provided timely (33). To alleviate the systemic inflammatory response, glucocorticoids can be used for severe ARDS patients, which can reduce the mortality rate (34). Additionally, sedatives should only be used in the early stages of patients with moderate-to-severe ARDS, and when there is still agitation and human–machine confrontation, muscle relaxants can be used (35). ARDS is a high-risk factor for deep vein thrombosis (DVT) and pulmonary embolism. Therefore, preventive anticoagulant therapy may be considered for ARDS patients without high-risk bleeding factors.

Early identification of the pathogen is crucial for targeted antibiotic treatment, which can reduce the mortality rate of ARDS caused by *C. psittaci*. However, for many hospitalized patients, timely and accurate diagnosis still poses challenges (36). Failure or delay in the diagnosis of infection may lead to prolonged hospital stays, readmission, and increased mortality rates (37, 38). In addition, undiagnosed patients always require empirical broad-spectrum antimicrobial therapy, increasing the risk of adverse drug reactions and antibiotic resistance (39). As a new approach to identify pathogens, mNGS has obvious advantages in detecting rare and complex pathogens in difficult cases, which is considered as a promising detection tool in the clinical diagnosis of unknown infections, such as *C. psittaci* (40). NTS is a fourth-generation sequencing technology that can provide faster and more comprehensive information for clinical diagnosis compared to mNGS. However, owing to its limitations, such as high cost and low popularity, there are few reports on its application in detecting *C. psittaci.* Although mNGS and NTS cannot replace traditional methods, they can serve as important supplements to microbiological pathogen testing tools and provide evidence for diagnosis in clinical. Among the 68 patients in our literature review, 64 were diagnosed with mNGS and 1 was diagnosed with NTS. There is no colonized *C. psittaci* in the normal human body, and *C. psittaci* belongs to intracellular bacteria. Due to the relatively small number of pathogens released into body fluids such as blood, sputum, and BALF, the detection sensitivity and positivity rate are relatively low. Therefore, once the DNA sequence of *C. psittaci* is detected, its potential as a pathogen must be considered. In our literature review, a patient with 8 DNA sequences of *C. psittaci* detected by mNGS still developed into a severe case (25). And in our case report, 70 DNA sequences of *C. psittaci* were detected in the BALF sample of the patient through mNGS. Therefore, even if a small amount of the *C. psittaci* DNA sequence was detected, it could be diagnosed as psittacosis, and targeted anti-infection treatment should be initiated in a timely manner.

*Chlamydia psittaci* is naturally resistant to *β*-lactams due to the lack of a cell wall, while tetracyclines and macrolides are effective drugs for treatment. Tetracyclines are regarded as the main recommended antibiotics for treating psittacosis. Among them, doxycycline is the preferred agent due to its strong intracellular activity and extensive clinical medication record (41). Over 90% of patients experience fever reduction within 48 h of taking doxycycline (42). In addition, minocycline has good antimicrobial activity and has been successfully applied in clinical practice (42). Tigecycline and omadacycline are the new generation of novel tetracycline antibiotics. Among them, tigecycline has a wide antibacterial spectrum and can be used as an alternative treatment for severe *C. psittaci* pneumonia, especially when patients are infected with other bacteria (43). Omadacycline has a higher concentration in the lungs than in the plasma and is eliminated mainly through feces. In patients with impaired liver and renal function and elderly individuals, the dosage does not need to be adjusted when omadacycline is used (44). However, owing to the immature clinical application of tigecycline and omadacycline, further large-scale clinical studies are still needed to observe whether their efficacy is superior to that of other tetracyclines. Macrolides, such as roxithromycin or azithromycin, can be used as alternative options for patients with a history of tetracycline allergy, as well as children, pregnant women, and other patients who are prohibited from using tetracyclines. *In vitro* studies have shown that fluoroquinolones are effective against *C. psittaci* (45), mainly by interfering with topoisomerase, thereby inhibiting the DNA synthesis of *C. psittaci*. However, the therapeutic effects of fluoroquinolones are not comparable to those of tetracyclines and macrolides (46), which may lead to treatment failure. At present, large-scale case–control studies comparing the efficacy differences between monotherapy and combination therapy are lacking. For patients with severe *C. psittaci* pneumonia or those with poor initial drug treatment, combination therapy may be preferable. In addition, antibiotic resistance is a clinical issue that needs attention. According to previous reports, genes related to the antibiotic resistance of *C. psittaci* include *16S rRNA*, *23S rRNA*, and *rpoB*, which are associated with different mechanisms leading to antibiotic resistance (47, 48). The abuse of antibiotics may further exacerbate the problem of microbial resistance, posing a threat to global public health safety. Therefore, precise targeted antimicrobial therapy is crucial.

Although the incidence rate of psittacosis is relatively low, it may lead to death in severe cases because of its strong pathogenicity, which poses a serious threat to human health. The nonspecific clinical manifestations and the lack of conventional detection methods pose challenges in the diagnosis of psittacosis. The contact history of birds and poultry before the onset of the disease has a suggestive effect on the diagnosis, and early identification needs to be combined with the actual medical history. Preliminary diagnosis can be quickly confirmed through mNGS, and once confirmed, targeted antimicrobial therapy should be administered as soon as possible. In addition, antibiotic resistance in Chlamydia remains a threat, and the development of new drugs against Chlamydia is urgently needed. At present, the prevention methods for psittacosis pneumonia are not yet mature, and it is necessary to explore new prevention methods or develop vaccines to prevent the spread and outbreak of this disease. Moreover, society and medical institutions need to attach great importance to the potential threat of *C. psittaci*, and it is recommended to include it in routine screening for respiratory pathogens. With the development of modern molecular biology technology and in-depth research on new drugs, more timely and accurate diagnostic methods for *C. psittaci* infection, as well as safer and more effective treatments, will provide strong support for clinical workers.

## 5 Conclusion

The clinical manifestations of *C. psittaci* pneumonia are diverse. For patients with severe *C. psittaci* pneumonia combined with ARDS, if not treated in a timely manner, it may be life-threatening. This study demonstrated the importance value of epidemiological investigations and the application of mNGS for early identification and diagnosis of psittacosis. As a highly promising detection tool, mNGS technology can help quickly and accurately identify pathogens, which contributes to promote the progress of targeted antimicrobial therapy and improve the prognosis of patients with severe infections. In addition, young psittacosis patients without underlying diseases should also be vigilant about the possibility of developing into severe cases.

## Data Availability

The original contributions presented in the study are included in the article/Supplementary material, further inquiries can be directed to the corresponding author.

## References

[1] Bachmann NL Polkinghorne A Timms P. Chlamydia genomics: providing novel insights into chlamydial biology. Trends Microbiol. (2014) 22:464–72. doi: 10.1016/j.tim.2014.04.013, PMID: 24882432

[2] Chan J Doyle B Branley J Sheppeard V Gabor M Viney K. An outbreak of psittacosis at a veterinary school demonstrating a novel source of infection. One Health. (2017) 3:29–33. doi: 10.1016/j.onehlt.2017.02.003, PMID: 28616500 PMC5454149

[3] Wallensten A Fredlund H Runehagen A. Multiple human-to-human transmission from a severe case of psittacosis, Sweden, January-February 2013. Euro Surveill. (2014) 19:20937. doi: 10.2807/1560-7917.es2014.19.42.20937, PMID: 25358043

[4] Zhang Z Zhou H Cao H Ji J Zhang R Li W. Human-to-human transmission of *Chlamydia psittaci* in China, 2020: an epidemiological and aetiological investigation. Lancet Microbe. (2022) 3:e512–20. doi: 10.1016/s2666-5247(22)00064-7, PMID: 35617977

[5] Liu S Cui Z Carr MJ Meng L Shi W Zhang Z. *Chlamydia psittaci* should be a notifiable infectious disease everywhere. Lancet Microbe. (2023) 4:e62–3. doi: 10.1016/s2666-5247(22)00306-8, PMID: 36372075

[6] Hogerwerf L de Gier B Baan B van der Hoek W. *Chlamydia psittaci* (psittacosis) as a cause of community-acquired pneumonia: a systematic review and meta-analysis. Epidemiol Infect. (2017) 145:3096–105. doi: 10.1017/s0950268817002060, PMID: 28946931 PMC9148753

[7] Wu X Li Y Zhang M Li M Zhang R Lu X. Etiology of severe community-acquired pneumonia in adults based on metagenomic next-generation sequencing: a prospective multicenter study. Infect Dis Ther. (2020) 9:1003–15. doi: 10.1007/s40121-020-00353-y, PMID: 33170499 PMC7652912

[8] Bellani G Laffey JG Pham T Fan E Brochard L Esteban A. Epidemiology, patterns of care, and mortality for patients with acute respiratory distress syndrome in intensive care units in 50 countries. JAMA. (2016) 315:788–800. doi: 10.1001/jama.2016.0291, PMID: 26903337

[9] Paolone S. Extracorporeal membrane oxygenation (ECMO) for lung injury in severe acute respiratory distress syndrome (ARDS): review of the literature. Clin Nurs Res. (2017) 26:747–62. doi: 10.1177/1054773816677808, PMID: 27836935

[10] Grasselli G Calfee CS Camporota L Poole D Amato MBP Antonelli M. ESICM guidelines on acute respiratory distress syndrome: definition, phenotyping and respiratory support strategies. Intensive Care Med. (2023) 49:727–59. doi: 10.1007/s00134-023-07050-7, PMID: 37326646 PMC10354163

[11] Shaw KA Szablewski CM Kellner S Kornegay L Bair P Brennan S. Psittacosis outbreak among Workers at Chicken Slaughter Plants, Virginia and Georgia, USA, 2018. Emerg Infect Dis. (2019) 25:2143–5. doi: 10.3201/eid2511.190703, PMID: 31625859 PMC6810211

[12] Yao W Chen X Wu Z Wang L Shi G Yang Z. A cluster of psittacosis cases in Lishui, Zhejiang Province, China, in 2021. Front Cell Infect Microbiol. (2022) 12:1044984. doi: 10.3389/fcimb.2022.1044984, PMID: 36590592 PMC9798449

[13] Beeckman DS Vanrompay DC. Zoonotic *Chlamydophila psittaci* infections from a clinical perspective. Clin Microbiol Infect. (2009) 15:11–7. doi: 10.1111/j.1469-0691.2008.02669.x, PMID: 19220335

[14] Burnard D Polkinghorne A. Chlamydial infections in wildlife-conservation threats and/or reservoirs of 'spill-over' infections? Vet Microbiol. (2016) 196:78–84. doi: 10.1016/j.vetmic.2016.10.018 27939160

[15] Dai N Li Q Geng J Guo W Yan W. Severe pneumonia caused by *Chlamydia psittaci*: report of two cases and literature review. J Infect Dev Ctries. (2022) 16:1101–12. doi: 10.3855/jidc.16166, PMID: 35797307

[16] Rehn M Ringberg H Runehagen A Herrmann B Olsen B Petersson AC. Unusual increase of psittacosis in southern Sweden linked to wild bird exposure, January to April 2013. Euro Surveill. (2013) 18:20478. doi: 10.2807/ese.18.19.20478-en, PMID: 23725809

[17] Branley J Bachmann NL Jelocnik M Myers GS Polkinghorne A. Australian human and parrot *Chlamydia psittaci* strains cluster within the highly virulent 6BC clade of this important zoonotic pathogen. Sci Rep. (2016) 6:30019. doi: 10.1038/srep30019, PMID: 27488134 PMC4973220

[18] Wang L Shi Z Chen W Du X Zhan L. Extracorporeal membrane oxygenation in severe acute respiratory distress syndrome caused by *Chlamydia psittaci*: a case report and review of the literature. Front Med. (2021) 8:731047. doi: 10.3389/fmed.2021.731047, PMID: 34722571 PMC8554049

[19] Ni Y Zhong H Gu Y Liu L Zhang Q Wang L. Clinical features, treatment, and outcome of psittacosis pneumonia: a multicenter study. Infect Dis. (2023) 10:ofac518. doi: 10.1093/ofid/ofac518, PMID: 36817742 PMC9937045

[20] Palmer SR. Psittacosis in man – recent developments in the UK: a review. J R Soc Med. (1982) 75:262–7. doi: 10.1177/014107688207500412, PMID: 7200143 PMC1437651

[21] Rybarczyk J Versteele C Lernout T Vanrompay D. Human psittacosis: a review with emphasis on surveillance in Belgium. Acta Clin Belg. (2020) 75:42–8. doi: 10.1080/17843286.2019.1590889, PMID: 30882289

[22] Katsura D Tsuji S Kimura F Tanaka T Eguchi Y Murakami T. Gestational psittacosis: a case report and literature review. J Obstet Gynaecol Res. (2020) 46:673–7. doi: 10.1111/jog.14217, PMID: 32077210

[23] Tantengco OAG. Gestational psittacosis: an emerging infection. Lancet Microbe. (2022) 3:e728. doi: 10.1016/s2666-5247(22)00191-4, PMID: 35817065

[24] Marchese S Marchese G Paviglianiti G Lapi M Ottoveggio G Pipitone G. A pediatric case of *Chlamydia psittaci* caused severe acute respiratory distress syndrome (ARDS) in Italy. Ital J Pediatr. (2023) 49:107. doi: 10.1186/s13052-023-01497-6, PMID: 37649055 PMC10468848

[25] Zhao ZL Tang X He CW Liu YL Li XY Wang R. Clinical characteristics and outcomes of acute respiratory distress syndrome caused by severe *Chlamydia psittaci* pneumonia. Zhonghua Jie He He Hu Xi Za Zhi. (2022) 45:1015–21. doi: 10.3760/cma.j.cn112147-20220221-00139, PMID: 36207958

[26] Cheong HC Lee CYQ Cheok YY Tan GMY Looi CY Wong WF. Chlamydiaceae: diseases in primary hosts and zoonosis. Microorganisms. (2019) 7:146. doi: 10.3390/microorganisms7050146, PMID: 31137741 PMC6560403

[27] Vanrompay D Ducatelle R Haesebrouck F Hendrickx W. Primary pathogenicity of an European isolate of *Chlamydia psittaci* from Turkey poults. Vet Microbiol. (1993) 38:103–13. doi: 10.1016/0378-1135(93)90078-l, PMID: 8128594

[28] Smith KA Bradley KK Stobierski MG Tengelsen LA. Compendium of measures to control *Chlamydophila psittaci* (formerly *Chlamydia psittaci*) infection among humans (psittacosis) and pet birds, 2005. J Am Vet Med Assoc. (2005) 226:532–9. doi: 10.2460/javma.2005.226.532, PMID: 15742693

[29] Meijer R Van Biezen P Prins G Boiten HJ. Multi-organ failure with necrotic skin lesions due to infection with *Chlamydia psittaci*. Int J Infect Dis. (2021) 106:262–4. doi: 10.1016/j.ijid.2021.03.091, PMID: 33823280

[30] Zhang H Zhan D Chen D Huang W Yu M Li Q. Next-generation sequencing diagnosis of severe pneumonia from fulminant psittacosis with multiple organ failure: a case report and literature review. Ann Transl Med. (2020) 8:401. doi: 10.21037/atm.2020.03.17, PMID: 32355845 PMC7186658

[31] Liu K Wu L Chen G Zeng D Zhong Q Luo L. Clinical characteristics of *Chlamydia psittaci* infection diagnosed by metagenomic next-generation sequencing: a retrospective multi-center study in Fujian, China. Infect Drug Resist. (2024) 17:697–708. doi: 10.2147/idr.S443953, PMID: 38405056 PMC10894596

[32] Fan E Del Sorbo L Goligher EC Hodgson CL Munshi L Walkey AJ. An official American Thoracic Society/European Society of Intensive Care Medicine/Society of Critical Care Medicine clinical practice guideline: mechanical ventilation in adult patients with acute respiratory distress syndrome. Am J Respir Crit Care Med. (2017) 195:1253–63. doi: 10.1164/rccm.201703-0548ST, PMID: 28459336

[33] Guo Y Cheng J Li Y. Influence of enteral nutrition initiation timing on curative effect and prognosis of acute respiratory distress syndrome patients with mechanical ventilation. Zhonghua Wei Zhong Bing Ji Jiu Yi Xue. (2018) 30:573–7. doi: 10.3760/cma.j.issn.2095-4352.2018.06.014, PMID: 30009734

[34] Chaudhuri D Nei AM Rochwerg B Balk RA Asehnoune K Cadena RS. Executive summary: guidelines on use of corticosteroids in critically ill patients with Sepsis, acute respiratory distress syndrome, and community-acquired pneumonia focused update 2024. Crit Care Med. (2024) 52:833–6. doi: 10.1097/ccm.0000000000006171, PMID: 38240490

[35] Moss M Huang DT Brower RG Ferguson ND Ginde AA Gong MN. Early neuromuscular blockade in the acute respiratory distress syndrome. N Engl J Med. (2019) 380:1997–2008. doi: 10.1056/NEJMoa1901686, PMID: 31112383 PMC6741345

[36] Messacar K Parker SK Todd JK Dominguez SR. Implementation of rapid molecular infectious disease diagnostics: the role of diagnostic and antimicrobial stewardship. J Clin Microbiol. (2017) 55:715–23. doi: 10.1128/jcm.02264-16, PMID: 28031432 PMC5328439

[37] Glimaker M Johansson B Grindborg Ö Bottai M Lindquist L Sjolin J. Adult bacterial meningitis: earlier treatment and improved outcome following guideline revision promoting prompt lumbar puncture. Clin Infect Dis. (2015) 60:1162–9. doi: 10.1093/cid/civ011, PMID: 25663160

[38] Weiss SL Fitzgerald JC Balamuth F Alpern ER Lavelle J Chilutti M. Delayed antimicrobial therapy increases mortality and organ dysfunction duration in pediatric sepsis. Crit Care Med. (2014) 42:2409–17. doi: 10.1097/ccm.0000000000000509, PMID: 25148597 PMC4213742

[39] Llor C Bjerrum L. Antimicrobial resistance: risk associated with antibiotic overuse and initiatives to reduce the problem. Ther Adv Drug Saf. (2014) 5:229–41. doi: 10.1177/2042098614554919, PMID: 25436105 PMC4232501

[40] Gu W Deng X Lee M Sucu YD Arevalo S Stryke D. Rapid pathogen detection by metagenomic next-generation sequencing of infected body fluids. Nat Med. (2021) 27:115–24. doi: 10.1038/s41591-020-1105-z, PMID: 33169017 PMC9020267

[41] Stewardson AJ Grayson ML. Psittacosis. Infect Dis Clin N Am. (2010) 24:7–25. doi: 10.1016/j.idc.2009.10.003 20171542

[42] Yung AP Grayson ML. Psittacosis--a review of 135 cases. Med J Aust. (1988) 148:228–33. doi: 10.5694/j.1326-5377.1988.tb99430.x 3343952

[43] Liu J Gao Y. Tigecycline in the treatment of severe pneumonia caused by *Chlamydia psittaci*: a case report and literature review. Front Med. (2022) 9:1040441. doi: 10.3389/fmed.2022.1040441, PMID: 36507520 PMC9730873

[44] Wang DX Xiao LX Deng XY Deng W. Omadacycline for the treatment of severe pneumonia caused by *Chlamydia psittaci* complicated with acute respiratory distress syndrome during the COVID-19 pandemic. Front Med. (2023) 10:1207534. doi: 10.3389/fmed.2023.1207534, PMID: 38264056 PMC10805100

[45] Donati M Rodrìguez Fermepin M Olmo A D'apote L Cevenini R. Comparative in-vitro activity of moxifloxacin, minocycline and azithromycin against Chlamydia spp. J Antimicrob Chemother. (1999) 43:825–7. doi: 10.1093/jac/43.6.825, PMID: 10404322

[46] Wu HH Feng LF Fang SY. Application of metagenomic next-generation sequencing in the diagnosis of severe pneumonia caused by *Chlamydia psittaci*. BMC Pulm Med. (2021) 21:300. doi: 10.1186/s12890-021-01673-6, PMID: 34556069 PMC8461849

[47] Benamri I Azzouzi M Sanak K Moussa A Radouani F. An overview of genes and mutations associated with Chlamydiae species' resistance to antibiotics. Ann Clin Microbiol Antimicrob. (2021) 20:59. doi: 10.1186/s12941-021-00465-4, PMID: 34479551 PMC8414684

[48] Lu H Yuan J Wu Z Wang L Wu S Chen Q. Distribution of drug-resistant genes in alveolar lavage fluid from patients with psittacosis and traceability analysis of causative organisms. Front Microbiol. (2023) 14:1182604. doi: 10.3389/fmicb.2023.1182604, PMID: 37425996 PMC10327639

[49] Wichert A Lukasewitz P Häuser MH Bittersohl J Lennartz H. ARDS in fulminant ornithosis and treatment with extracorporeal lung assist. Int J Artif Organs. (2000) 23:371–4. doi: 10.1177/039139880002300605, PMID: 10919754

[50] Yang F Li J Qi B Zou L Shi Z Lei Y. Clinical symptoms and outcomes of severe pneumonia caused by *Chlamydia psittaci* in Southwest China. Front Cell Infect Microbiol. (2021) 11:727594. doi: 10.3389/fcimb.2021.727594, PMID: 35071027 PMC8770948

[51] Ferreira VL Silva MV Bassetti BR Pellini ACG Raso TF. Intersectoral action for health: preventing psittacosis spread after one reported case. Epidemiol Infect. (2017) 145:2263–8. doi: 10.1017/s0950268817001042, PMID: 28554339 PMC9148848

[52] Yang SL Gao Y Han ZY Du X Liu W Jin SG. Successful treatment of near-fatal pulmonary embolism and cardiac arrest in an adult patient with fulminant psittacosis-induced severe acute respiratory distress syndrome after veno-venous extracorporeal membrane oxygenation rescue: a case report and follow-up. Heliyon. (2023) 9:e20562. doi: 10.1016/j.heliyon.2023.e20562, PMID: 37842616 PMC10568334

[53] Tang J Tan W Luo L Xu H Li N. Application of metagenomic next-generation sequencing in the diagnosis of pneumonia caused by *Chlamydia psittaci*. Microbiol Spectr. (2022) 10:e0238421. doi: 10.1128/spectrum.02384-21, PMID: 35938720 PMC9431268

