# Supplementary material for: Acute respiratory distress syndrome caused by Chlamydia psittaci: a case report and literature review
Source: Front Med (Lausanne). 2024 Dec 3;11:1418241. doi: 10.3389/fmed.2024.1418241 (PMC11653191; doi:10.3389/fmed.2024.1418241)
Supplement: Supplementary file 1 [file Data_Sheet_1.pdf]

Table S1. Comparison of the Survived and Deceased Patients in our literature review.

Variables	No. (%) of cases		*P* value
	Survived group (n=62)	Deceased group (n=6)	
**Clinical symptoms on the day of admission**			
Cough	32(51.6)	5(83.3)	0.209
Fever	57(91.9)	5(83.3)	0.438
Dyspnea	43(69.4)	5(83.3)	0.662
Headache	16(25.8)	2(33.3)	0.652
Diarrhea	4(6.5)	0(0.0)	1.000
Backache	1(1.6)	0(0.0)	1.000
Myalgia	10(16.1)	1(16.7)	1.000
Chest pain	2(3.2)	0(0.0)	1.000
Confusion	2(3.2)	0(0.0)	1.000
Hemoptysis	4(6.5)	0(0.0)	1.000
Chills	1(1.6)	0(0.0)	1.000
Shiver	5(8.1)	1(16.7)	0.438
Weakness	10(16.1)	0(0.0)	0.581
Poor appetite	10(16.1)	0(0.0)	0.581
**Complications**			
Hepatic dysfunction	52(83.9)	6(100.0)	0.581
Renal dysfunction	8(12.9)	5(83.3)	**0.001**
Neurological symptoms	9(14.5)	3(50.0)	0.063
Cardiac damage	17(27.4)	2(33.3)	1.000
Gastrointestinal symptoms	3(4.8)	0(0.0)	1.000
Pulmonary embolism	8(12.9)	0(0.0)	1.000
**ECMO**	5(8.1)	0(0.0)	1.000
